# Measuring the relationship between social capital, race, and education

**DOI:** 10.3389/fpubh.2025.1632838

**Published:** 2025-10-17

**Authors:** Jennifer Contreras, Christopher M. Amissah, Abdolvahab Khademi, Christine Valeriann, Ester Villalonga-Olives

**Affiliations:** Department of Practice, Sciences, and Health Outcomes Research, University of Maryland School of Pharmacy, Baltimore, MD, United States

**Keywords:** social capital, social connectedness, community, race, education, longitudinal, disparities

## Abstract

Enhancing social and community support (e.g., social capital) is essential for building healthier communities, as social capital significantly influences health outcomes. However, the relationship between social capital, race, and education is complex. Historically marginalized groups often face systemic barriers that reduce their social capital. Therefore, longitudinal research is essential to understand these dynamics and address health disparities. This study explores the relationship between social capital, race, and education in U.S. adults over time, using Midlife in the U.S. (MIDUS) data from Waves 1–3 (1995–1996; 2004–2006; 2013–2014). We used the disparity assessment framework from Ward et al. and multilevel mixed-effects models to investigate how social capital evolved differently based on race and education as well as the potential implications of these differences. Our findings revealed that Black respondents consistently demonstrated higher community contributions and community involvement compared with White respondents, despite having lower education on average. This social capital advantage for Black respondents persisted across all three waves of the MIDUS study. Longitudinal analysis also showed that community contributions remained stable at all time points for all respondents, while community involvement declined at MIDUS 3. However, Black respondents exhibited a prominent increase in community involvement at MIDUS 3, suggesting that Black communities may have adapted and thrived through culturally specific forms of social capital during that period. Our findings indicated these positive manifestations of social capital should be explored to see how it can be supported and suggested the need for further exploration of racial dynamics and culturally specific forms of social capital.

## 1 Introduction

Healthy People 2030 is a national initiative led by the U.S. Department of Health and Human Services (HHS) to improve the health and wellbeing of all Americans. Agencies such as the Centers for Disease Control and Prevention and the National Institutes of Health have incorporated Healthy People 2030 objectives into their programs and policies. Healthy People 2030 places a strong emphasis on social determinants of health and social needs (1). Therefore, addressing these factors has the potential to reduce health inequities and build healthier communities by enhancing social and community support systems (2). Social and community support encompasses social capital, which involves resources such as trust, community involvement, cooperation, and information connected to social networks and relationships (3, 4). Increasing social and community support is essential for promoting healthier communities because it addresses critical factors that influence overall health and wellbeing such as improving access to key resources, encouraging healthy behaviors, building resilience, and enhancing social cohesion (5–9).

When an individual has close relationships with family and friends, it provides social support and enhances social capital. Additionally, having a social network with many supportive connections can also make meaningful contributions to social capital, both personally and within the community (10–12). Hence, social capital plays a vital role in shaping the health of communities and all their participants (12–14). When individuals actively engage in community activities and contribute to the collective wellbeing of others, they establish meaningful social connections and support networks. In turn, these social ties contribute significantly to one’s physical, emotional, and psychological wellbeing (6, 9, 13). For example, social capital has been associated with improving several health outcomes, such as obesity, diabetes, depression, cardiovascular disease, cancer, and all-cause mortality (15–20, 40).

Another way social capital influences health outcomes is through the presence of networks that may encourage either health-promoting or health-compromising behaviors. For instance, being part of a network that normalizes binge drinking or other risky behaviors can negatively impact health (4). Conversely, networks that emphasize wellbeing can support healthier choices, offer emotional and practical support, and enhance access to care and resources. The collective strength of supportive networks helps reduce stress and fosters initiatives that improve wellbeing, leading to lower rates of diabetes, depression, cardiovascular and kidney disease, as well as obesity (7–9, 19). Some evidence suggests that interventions focusing on social capital (and more broadly on social connectedness) have helped underserved communities with limited access to health care (6, 21–23, 41, 44). These interventions often help members have more access to community and health care resources as well as promote community engagement, leading to reduced mental health symptoms and improved overall wellbeing.

Historically, racial disparities in access to quality education and opportunities have perpetuated social inequalities (24, 25). In a review by Gilbert et al. (25), the authors explained that Black people in the U.S. faced systemic barriers limiting their education, which hindered their ability to acquire the necessary skills and knowledge for social capital accumulation (24, 25). For example, disparities in education contributed to unequal access to social networks, professional opportunities, and influential connections that were crucial for social capital and social mobility (25, 26). Notably, both education and race play an important role in social capital, with studies revealing that Black populations often have less robust social networks due to educational and work settings (27, 28, 42). Therefore, understanding the relationship between race, education, and social capital is critical for addressing systemic inequality and promoting healthy communities.

Only a few investigations have begun to uncover the complex relationship between social capital and race (29–31). For example, Hutchinson et al. (29) examined the relationship between neighborhood racial composition, social capital, and Black all-cause mortality in Philadelphia, finding that higher neighborhood social capital was associated with lower mortality among Black participants. Similarly, in a study by Ransome et al. (31), the relationship between social capital and health varied by race, showing that greater trust in neighbors was linked to a reduced likelihood of undergoing HIV testing. This inverse association was stronger among Hispanic/Latino and White individuals compared with Black individuals. These studies have been foundational to observe differences in social capital by education and race. Yet, what remains unknown is the longitudinal relationship between social capital, race, and education.

The purpose of our study was to examine the longitudinal differences in social capital across various racial groups and levels of education. By investigating these disparities over time, we aimed to understand how social capital evolved differently based on race and education as well as the potential implications of these differences to inform the development of interventions and programs promoting social capital and reducing disparities.

## 2 Methods

### 2.1 Data source

We used the publicly available longitudinal data from the Midlife in the U.S. (MIDUS) study, an ongoing national longitudinal study led by the University of Wisconsin-Madison Institute on Aging. The purpose of MIDUS is to examine the health of the U.S. adult population (43), specifically, the role of behavioral, psychological, and social factors on age-related variations in the health and wellbeing of adults.

The first wave of the MIDUS study (MIDUS 1) was conducted using survey data collected during 1995–1996 from a sample of 7,000 U.S. adults aged 25–74. For the second wave (MIDUS 2), data was collected between 2004 and 2005, with an expanded scope to include cognition data and daily experiences, along with a new sample of Black respondents from Milwaukee, WI. The third wave (MIDUS 3) collected data between 2013 and 2022. MIDUS 3 included a core survey data, and a retention-early warning component aimed at reinstating participants who had previously dropped out. The retention of the sample in the MIDUS longitudinal study remained high. Of the original MIDUS 1 (1995–1996) sample, 69% of the cognitive interview participants returned for MIDUS 2 (2004–2005) and 77% (adjusted for mortality) participated in MIDUS 3 (2013–2014) (43).

We included national respondents who completed all three waves in our study (2,264 White respondents and 76 Black respondents). We used multiple imputation to address missing data by generating several plausible values for each missing observation and creating multiple complete datasets.

### 2.2 Measures

#### 2.2.1 Social capital measures

We used two social capital measures: “Contributions to Community” and “Community Involvement” scales. The “Contributions to Community” scale measures how much the respondent contributes to their community in various forms. This scale measures the extent to which respondents perceive themselves as making meaningful contributions to their community, based on six indicators: offering unique contributions, possessing transferable skills, being sought out for advice, feeling needed, having a positive influence on others, and a willingness to teach or share knowledge. Response scales range from “A lot” (1) to “Not at all” (4). The responses for this scale was reverse coded so that higher scores reflected greater community contributions.

The “Community Involvement” scale measures the extent to which the participant is involved in their community. The “Community Involvement” scale includes five key indicators: the past, present, and anticipated future contributions to the wellbeing of others; perceived control over making these contributions; and the amount of thought and effort respondents’ invest in them. Responses are rated on a 0–10 scale, with 0 being the lowest contribution and 10 being the highest contribution.

#### 2.2.2 Social demographic characteristics

For predicting measures of social capital, we selected two sociodemographic characteristics from respondents—race and education. The race variable included Black and White categories. The education variable included four category levels: “Less than high school education,” “High school graduate or GED,” “Some college education,” and “College graduate or higher.”

### 2.3 Analysis

We supplemented the MIDUS data to increase the sample size of Black respondents and performed multiple imputations to address for missingness in the data. Given the absence of psychometric tests evaluating the structure of the “Contributions to Community” and “Community Involvement” scales, we conducted a one-factor confirmatory factor analysis (CFA) using the *lavaan* package in R version 4.3.3 (32, 33). As part of the CFA, we assessed the fit of the hypothesized measurement model for the scales using the Comparative Fit Index (CFI), Tucker-Lewis Index (TLI), and Root Mean Square Error of Approximation (RMSEA). An acceptable model fit was indicated by CFI and TLI values > 0.90 and an RMSEA value < 0.05 (34).

We modeled longitudinal data for MIDUS 1 (1995–1996), MIDUS 2 (2004–2006), and MIDUS 3 (2013–2014) using multilevel linear mixed-effects modeling where measurements $Y_{\mathrm{ti}}$ at each time point *t* is nested in individual *i* and using the *lmer* function from the *lme4* package in *R*, which is specialized for fitting linear mixed-effects models (33). We used the fixed effects of race and education as predictors for “Contributions to Community” and “Community Involvement.” For the mixed-effects model, we designated “White” as the reference category for race and “less than high school education” as the reference category for education. This allowed us to compare the impacts of being Black and other education categories against these reference categories. We used a random intercept model to investigate the relationships among these variables. The equation for the model is below:

$$Y_{\mathrm{ti}}=\pi_{0i}+\pi_{1i}t_{i}+\varepsilon_{\mathrm{ti}}$$

Where:

$$\begin{array}{l} \pi_{0i}=\beta_{00}+\beta_{01}(\text{Race})+\beta_{02}(\mathrm{Edu})+\beta_{03}(\text{Time})\\ +\beta_{04}(\text{Race}\times \mathrm{Edu})+\beta_{05}(\text{Race}\times \text{Time})+\\ \;\;\;\;\;\;\;\;\;\beta_{06}(\mathrm{Edu}\times \text{Time})+\beta_{07}(\text{Race}\times \mathrm{Edu}\times \text{Time})+u_{0i} \end{array}$$

$$\begin{array}{l} \pi_{1i}=\beta_{10}+\beta_{11}(\text{Race})+\beta_{12}(\mathrm{Edu})+\beta_{13}(\text{Time})\\ +\beta_{14}(\text{Race}\times \mathrm{Edu})+\beta_{15}(\text{Race}\times \text{Time})+\beta_{16}\\ \;\;\;\;\;\;\;\;\;\;\;\;(\mathrm{Edu}\times \text{Time})+\beta_{17}(\text{Race}\times \mathrm{Edu}\times \text{Time}) \end{array}$$

Where:

-
$Y_{\mathrm{ti}}$ is the outcome for individual *i* at time *t*
-
$\pi_{0i}$ is the random intercept for individual *i*, the baseline value of the outcome for the individual
-
$\pi_{1i}$ is the random slope for individual *i*, assessing changes in the outcome over time for the individual
-
$t_{i}$ represents the time variable for individual *i*, covering three discrete time points (MIDUS 1–3)
-
$\varepsilon_{\mathrm{ti}}$ is the residual error, which captures unexplained variance at the individual level at time *t*, after accounting for both fixed and random effects
-
β is the fixed effects, including intercepts and slopes for the predictors and their interactions
-
$u_{0i}$ represents a random intercept which allows each group to have its own baseline value

#### 2.3.1 Application of the comprehensive framework for assessing disparities

To enhance the rigor of the investigation we employed Ward et al.’ (35) framework for assessing health disparities. This is a comprehensive framework for disparity investigation beyond the evaluation of significant interactions, which examines group-specific differences in outcome prevalence, exposure prevalence, and effect size.

In health disparities research, relying on the interpretation of an interaction term can be limiting because it may oversimplify complex relationships between exposure, outcome, and subgroup characteristics (35). Interaction terms typically indicate statistical interaction but do not always provide insight into underlying mechanisms or whether observed differences are meaningful in real-world settings. Additionally, focusing exclusively on interaction terms might overlook the broader context of social determinants, structural factors, or cultural influences that contribute to disparities. To overcome this limitation, Ward and colleagues suggest that researchers consider the following questions: (1) *Is there a difference in the outcome between groups?* (2) *Is there a difference in the exposure between groups?* and (3) *Does the effect of the exposure on the outcome differ between groups?* This approach enables researchers to identify whether observed differences in health outcomes are due to exposure differences, variations in exposure-outcome associations, or genuine disparities in response to the exposure itself. By using this framework, we were able to more comprehensively assess disparities and move beyond statistical interactions to a nuanced understanding of how exposure effects vary across groups.

## 3 Results

We begin by presenting the results of the CFA and key descriptive statistics. We then explore longitudinal trends in social capital to highlight emerging patterns over time. Finally, we address the three guiding questions posed by Ward et al. (35) by integrating findings from the mixed-effects models and visual comparisons using boxplots to examine differences across racial groups.

### 3.1 Confirmatory factor analysis

One-factor model fit results for the *Contributions to Community* and *Community Involvement* scales demonstrated excellent and acceptable fit, respectively. The CFA one-factor model for *Contributions to Community* produced a CFI value of 0.98, TLI value of 0.98, and an RMSEA value of 0.03. For *Community Involvement*, the CFA one factor model produced a CFI value of 0.91, a TLI value of 0.90, and an RMSEA value of 0.05.

### 3.2 Racial differences in community engagement over time

**Figure 1:**
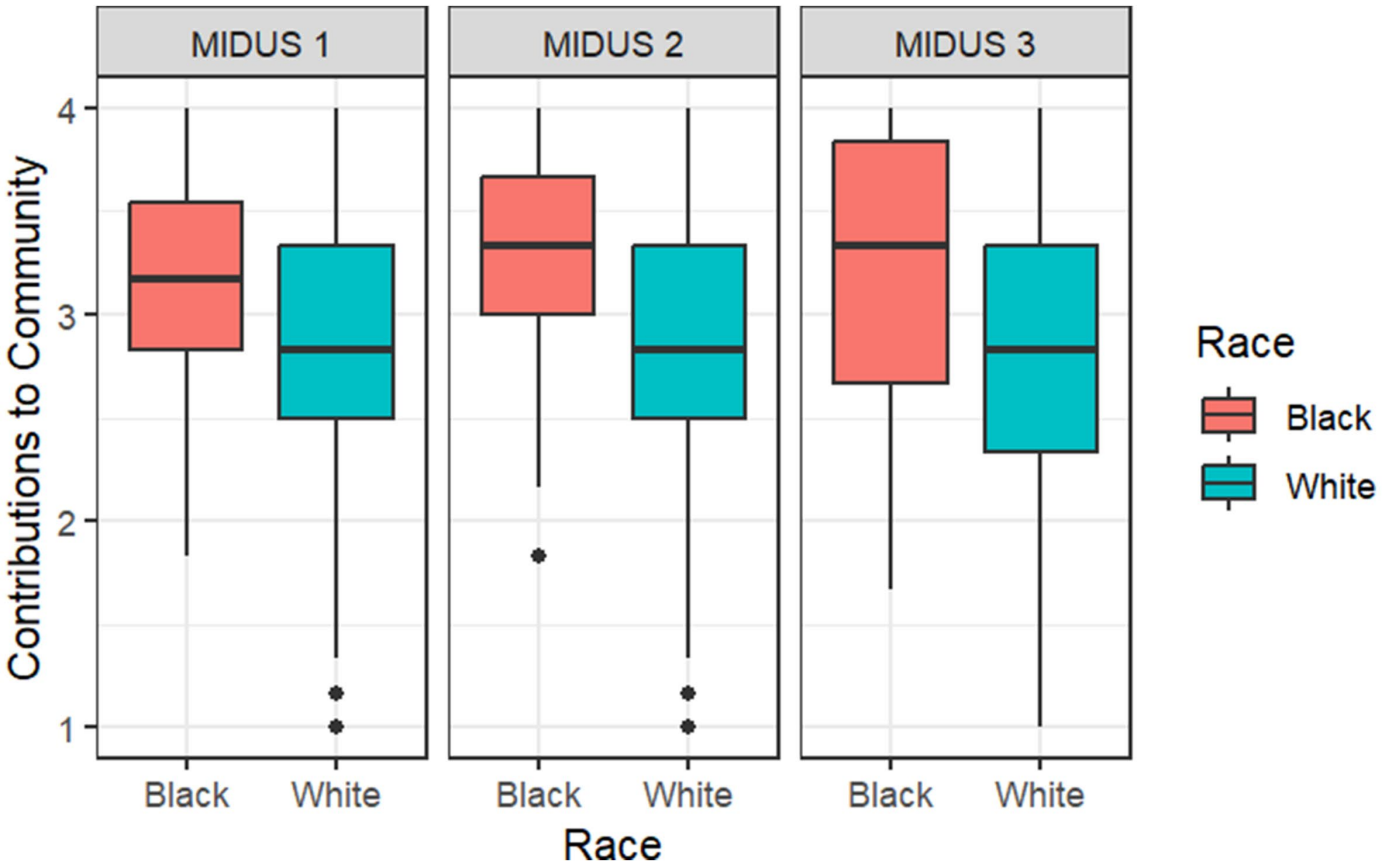
Racial differences in “Contributions to Community” across time. Median score is indicated by the line inside the box; Interquartile range is indicated by the length of the box; Range is indicated by the whiskers; Outliers are indicated by points beyond the whiskers. A wider box or longer whiskers for one group suggests a greater variability in scores, and a higher median indicates greater “Contributions to Community.” The total sample size for this analysis is *N* = 2,324.

**Figure 2:**
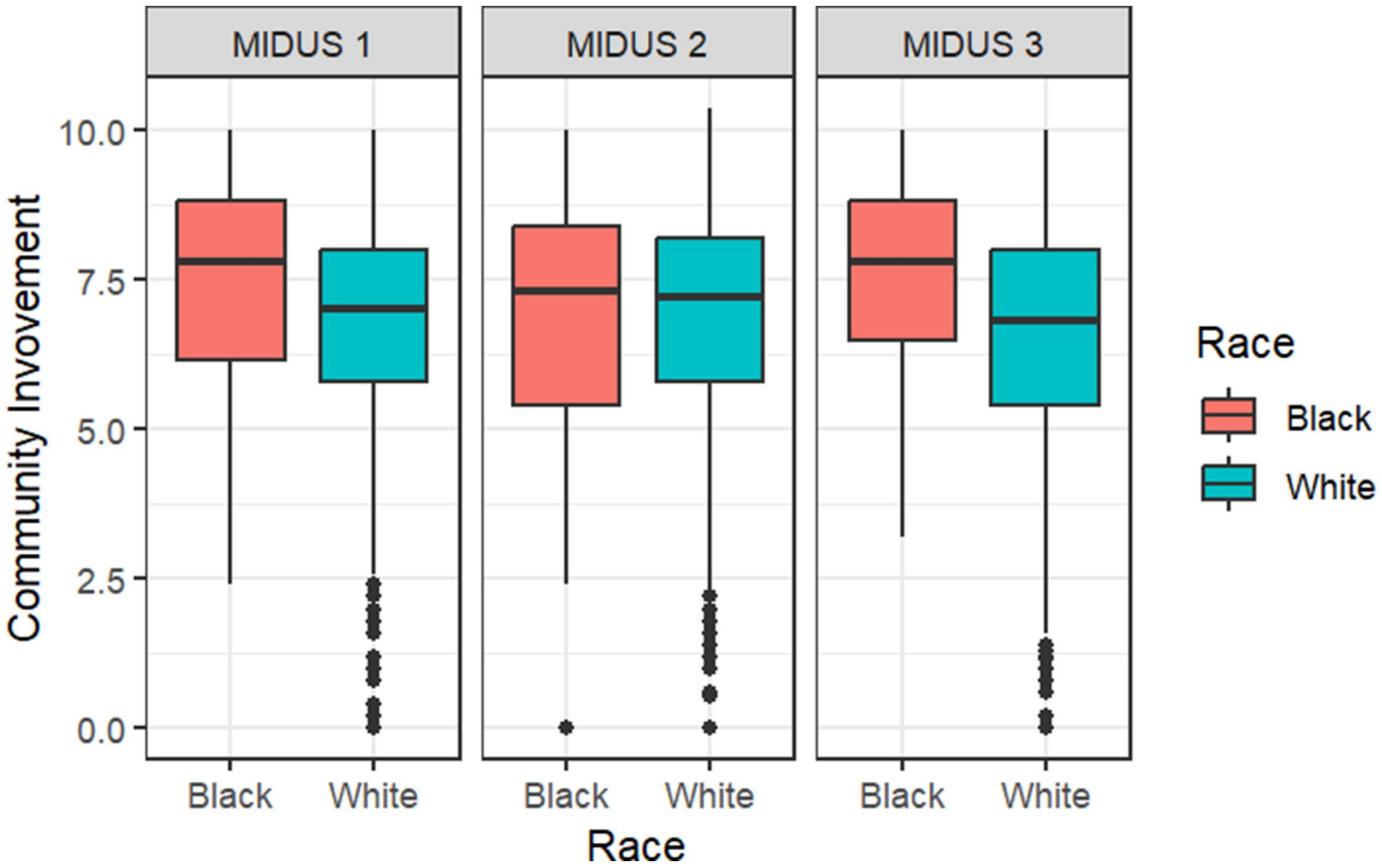
Racial differences in “Community Involvement” across time. Median score is indicated by the line inside the box; Interquartile range is indicated by the length of the box; Range is indicated by the whiskers; Outliers are indicated by points beyond the whiskers. A wider box or longer whiskers for one group suggests a greater variability in scores, and a higher median indicates higher “Community Involvement.” The total sample size for this analysis is *N* = 2,481.

Figures 1, 2 assess racial differences in “Contributions to Community” and “Community Involvement” across three different time points: MIDUS 1 (1995–1996), MIDUS 2 (2004–2006), and MIDUS 3 (2013–2014). For each racial group, the plot shows the median score (the line inside the box), the interquartile range (the length of the box), and the range of the data (indicated by the whiskers). The presence of outliers is also highlighted beyond the whiskers. From the plots, we can compare the median scores and spread of “Contributions to Community” and “Community Involvement” between the two racial groups. A wider box or longer whiskers for one group suggests a greater variability in scores, and a higher median indicates greater community contributions or higher involvement. Differences in the position of the medians and the spread of the boxes across groups suggest racial disparities in community contributions and involvement. Figure 1 shows that Black respondents had higher median scores than White respondents on all three time points, suggesting that Black respondents contributed more to their community than their White counterparts. Figure 2 shows that Black respondents had higher median scores than White respondents on MIDUS 1 (1995–1996) and MIDUS 3 (2013–2014), pointing to their higher level of community involvement. In short, both Figures 1, 2 show that race-specific “Contributions to Community” and “Community Involvement” were relatively stable over time.

### 3.3 Longitudinal trends in social capital

The longitudinal analyses of time in the mixed-effects model (Table 1) revealed that overall “Contributions to Community” remained stable over time (*β*_MIDUS 2_ = −0.01, *p* > 0.05; *β*_MIDUS 3_ = −0.10, *p* > 0.05), but there was a significant reduction in “Community Involvement” at MIDUS 3 (*β*_MIDUS 2_ = −0.29, *p* > 0.05; *β*_MIDUS 3_ = −1.10, *p* < 0.001). Similarly, race-specific “Contributions to Community” remained stable over time (*β*_Black * MIDUS 2_ = −0.05, *p* > 0.05; *β*_Black * MIDUS 3_ = −0.15, *p* > 0.05), but there was a significant increase in “Community Involvement” among Black respondents at MIDUS 3 (*β*_Black * MIDUS 2_ = 0.51, *p* > 0.05; *β*_Black * MIDUS 3_ = 1.92, *p* = 0.019). Also, time and education did not have any significant interaction effect on “Contributions to Community” at either MIDUS 2 or MIDUS 3 (*p* > 0.05), suggesting that contributions remained stable across time irrespective of the participant’s educational background. However, the interaction effects of time and education on “Community Involvement” were significant at MIDUS 3 (*β*_High school diploma or GED * MIDUS 3_ = 0.70, *p* = 0.008; *β*_Some college education * MIDUS 3_ = 0.64, *p* = 0.013; *β*_College degree or higher * MIDUS 3_ = 1.07, *p* < 0.001), pointing to a greater involvement in community at higher educational levels at MIDUS 3 (2013–2014) compared with MIDUS 1 (1995–1996).

**Table 1:** “Contributions to Community” and “Community Involvement” by White and Black respondents with different educational levels across time.

Fixed effects	“Contributions to Community”			“Community Involvement”		
	Estimate (*β*)	S.E.	*p*-value	Estimate (*β*)	S.E.	*p*-value
(Intercept)	2.52	0.09	<0.001^***^	6.56	0.23	<0.001^***^
Black	0.73	0.27	0.007^**^	0.71	0.76	0.346
High school diploma or GED	0.16	0.09	0.095	0.05	0.24	0.827
Some college education	0.29	0.09	0.002^**^	0.30	0.24	0.217
College degree or higher	0.46	0.09	<0.001^***^	0.43	0.24	0.068
MIDUS 2	−0.01	0.08	0.897	−0.29	0.25	0.248
MIDUS 3	−0.10	0.08	0.210	−1.10	0.25	<0.001^***^
Black × High school diploma or GED	−0.31	0.34	0.354	0.02	0.89	0.982
Black × Some college education	−0.64	0.31	0.038^*^	−0.25	0.84	0.771
Black × College degree or higher	−0.47	0.29	0.109	−0.18	0.82	0.828
Black × MIDUS 2	−0.05	0.24	0.851	0.51	0.82	0.530
Black × MIDUS 3	−0.15	0.24	0.535	1.92	0.82	0.019^*^
High school diploma or GED × MIDUS 2	0.03	0.08	0.736	0.40	0.26	0.130
Some college education × MIDUS 2	−0.02	0.08	0.835	0.21	0.26	0.423
College degree or higher × MIDUS 2	0.04	0.08	0.669	0.38	0.25	0.140
High school diploma or GED × MIDUS 3	0.05	0.08	0.540	0.70	0.26	0.008^**^
Some college education × MIDUS 3	0.01	0.08	0.932	0.64	0.26	0.013^*^
College degree or higher × MIDUS 3	0.06	0.08	0.429	1.07	0.25	<0.001^***^
Black × High school diploma or GED × MIDUS 2	0.31	0.31	0.317	−3.13	0.96	0.001^**^
Black × Some college education × MIDUS 2	0.19	0.28	0.482	−0.78	0.91	0.391
Black × College degree or higher × MIDUS 2	0.13	0.26	0.635	−0.43	0.89	0.625
Black × High school diploma or GED × MIDUS 3	0.44	0.31	0.150	−2.06	0.96	0.032^*^
Black × Some college education × MIDUS 3	0.40	0.28	0.145	−1.22	0.91	0.180
Black × College degree or higher × MIDUS 3	0.25	0.26	0.350	−1.57	0.89	0.077

In the regression model, each categorical predictor includes a baseline or reference level, which is omitted from the table as its estimate is assumed to be 0. These omitted levels serve as the comparison group for interpreting the effects of other categories. For the race variable, White is the reference group; for the time variable, MIDUS 1 is the reference group; and for the education variable, less than high school is the reference group.

^*^p < 0.05, ^**^p < 0.01, ^***^p < 0.001.

### 3.4 Outcomes across racial groups

#### 3.4.1 Is there a difference in the outcome between groups?

To address the first question in the framework by Ward et al., we examined racial differences in the two social capital outcome variables: “Contributions to Community” and “Community Involvement.” The mixed-effects model (Table 1) revealed a significant racial difference in “Contributions to Community” (*β*_Black_ = 0.73, *p* = 0.007), suggesting that being Black is associated with greater community contributions. Although the *p*-value indicated a lack of significant racial difference for “Community Involvement” (*β*_Black_ = 0.71, *p* > 0.05), the effect size (*β* = 0.71) was large enough to warrant further exploration of the existence of racial difference for the involvement of Black respondents (35). The boxplots in Figures 1, 2 support this observation, showing patterns that align with the large effect size.

#### 3.4.2 Is there a difference in exposure between groups?

To answer the second question, we compared the proportion of White and Black respondents across four levels of education using Fisher’s Exact Test with Monte Carlo simulation. We chose this approach due to the small sample size of Black respondents and the extreme imbalance in the expected frequency distributions between the White and Black samples across different education levels (see Table 2). The simulation involved 10,000 iterations to estimate the *p*-value. The results revealed a statistically significant difference in the racial distribution of respondents across the four education levels under “Contribution to Community” (*p* = 0.01) and “Community Involvement” (*p* = 0.07). This suggests that race is a factor influencing the distribution of education. For instance, in Table 2, for “Contributions to Community,” 10.0% of Black respondents had less than a high school education compared with 2.1% of White respondents. Also, 15.0% of Black respondents had a high school diploma or GED compared with 20.4% of White respondents. Similar patterns were observed for “Community Involvement.” These differences point to potential disparities in education between the two racial groups.

**Table 2:** Educational distribution among Black and White respondents for “Contributions to Community” and “Community Involvement.”

Education	“Contribution to Community”		“Community Involvement”	
	White *N* = 2,264 (%)	Black *N* = 60 (%)	White *N* = 2,405 (%)	Black *N* = 76 (%)
Less than high school education	48 (2.1%)	6 (10.0%)	59 (2.5%)	6 (7.9%)
High school diploma or GED	462 (20.4%)	9 (15.0%)	497 (20.7%)	15 (19.7%)
Some college education	680 (30.0%)	18 (30.0%)	726 (30.2%)	23 (30.3%)
College degree or higher	1,074 (47.4%)	27 (45.0%)	1,123 (46.7%)	32 (42.1%)

We conducted Fisher’s Exact Test with Monte Carlo simulation (10,000 iterations). “Contributions to Community” *p* = 0.01, “Community Involvement” *p* = 0.07.

Meanwhile, the mixed-effects model (Table 1) revealed a significant relationship between education on community contributions (*β*_Some college education_ = 0.29, *p* = 0.002, *β*_College degree or higher_ = 0.46, *p* < 0.001). Although the effect of education on community involvement was statistically not significant—probably due to the limitation in sample distribution—the corresponding effect sizes (βetas) were as large as those observed for community contributions. Differences in the position of the medians and the spread of the boxes across groups suggest potential disparities in community contributions or community involvement based on respondents’ educational levels. Overall, higher education groups had higher median scores, suggesting greater community contributions and higher community involvement.

#### 3.4.3 Does the effect of exposure on the outcome differ between groups?

Regarding the third question, the results of the mixed-effects model (Table 1) indicate that the interaction effects of race and education were not significant (*p* > 0.05), except for Black people with some college education, whose “Contributions to Community” scores were significantly lower than those of White respondents with less than high school education (*β*_Black people with some college education_ = −0.64, *p* = 0.038). The overall Black advantage in social capital—“Contributions to Community” and “Community Involvement”—was not affected by education. Black respondents maintained a social capital advantage over White respondents, regardless of education level.

## 4 Discussion

### 4.1 Longitudinal trends in social capital

The longitudinal analyses proved valuable and provided new insight into the stability and evolution of social capital over time. Findings suggest that an individual’s overall capacity or willingness to contribute to their community does not significantly change over time, regardless of racial group or education group. This implies that social capital is shaped by enduring individual factors (e.g., socioeconomic status) and structural factors (e.g., access to resources) rather than temporal shifts. Conversely, findings suggest that the reality of how an individual engages with their community does change over time. This implies that involvement varies as responsibilities (e.g., family or work obligations) and community dynamics (e.g., changes in social networks or local leadership) evolve. Furthermore, these changes in community involvement occurred in the later time period studied (i.e., the last wave of the study), suggesting that physical factors due to aging may also play a role (e.g., reduced mobility or declined health).

Importantly, the results strongly suggest that race is a powerful indicator of how community involvement changes over time, with White involvement significantly declining and Black involvement dramatically increasing in the respondent’s later years. This trend may be driven by the Black community’s generally heightened engagement in culturally specific forms of social capital, such as church activities, grassroots activism, or collective responses to societal challenges. This potentially offers Black people more support and adaptability than White people, who generally have lower engagement in culturally specific forms of social capital.

### 4.2 Racial differences in social capital

Race was strongly associated with social capital outcomes, particularly with higher community involvement over time. Black respondents demonstrated greater contributions and involvement than White respondents at most time points. This racial advantage may reflect longstanding cultural, familial, and institutional mechanisms such as church involvement, mutual aid networks, and collective action traditions that foster engagement irrespective of educational attainment.

### 4.3 Educational differences in social capital

Our study shows that education is a predictor of greater social capital. Participants with some college education or a college degree reported significantly greater contributions than those with less than a high school education. The significant interaction effects between education and time on community involvement at MIDUS 3 (Wave 3) suggest that the benefits of education on social capital accumulate or become more evident later in life. This demonstrates the role of education in fostering civic engagement and transferable skills that enhance long-term community participation.

### 4.4 Interaction effects

The significant interaction effects between time and education on community involvement at the later time period (i.e., higher levels of education were associated with greater increases in involvement) further emphasize the role of education in shaping social capital overall. This implies that education may provide individuals with the resources, networks, and/or skills necessary to participate more actively in their communities. Therefore, structural factors, such as access to education, play an important role in facilitating social capital development over time.

Our study produced inconclusive findings regarding race-by-education interactions. Most interaction effects were not significant, except for Black respondents with some college education, who reported lower community contributions compared to White respondents with less than high school education. This highlights a potential nuanced interaction between race and education that may reflect differing expectations (e.g., collective or public good) or personal experiences (e.g., participating in Black Greek organizations) within this subgroup. Black respondents maintained a social capital advantage across all other educational levels, suggesting that racial identity may play a critical role in shaping social capital. Their community behavior may be deeply rooted in historical and cultural factors, independent of formal education and attainment level.

### 4.5 Consistent racial advantage despite disparities

In our study, Black respondents were disproportionately represented in lower education categories, which underscores the persistent impact of historical and systemic inequities on educational opportunities. Results reflect the pattern of longstanding barriers in predominantly Black communities such as discriminatory policies, underfunded schools, and limited access to higher education resources. These educational disparities are not merely individual outcomes but are deeply rooted in structural inequalities that perpetuate cycles of disadvantage across generations.

Despite this overall educational disadvantage, Black respondents consistently exhibited higher community contributions and higher community involvement compared with their White counterparts. This racial advantage emphasizes the capacity of Black communities in fostering social capital, even in the face of disparities and systemic challenges. Black communities often leverage alternative pathways to develop and sustain social capital, such as strong familial bonds, church involvement, and mutual aid networks (25, 36). These mechanisms may compensate for—or even surpass—the contributions typically associated with higher education, reflecting a culturally rich and community-oriented approach to building and maintaining social capital.

Our findings revealed a racial disparity in community contributions and community involvement, with Black respondents demonstrating an advantage in both outcome variables compared to White respondents. In terms of exposure prevalence, Black respondents had lower education than White respondents. Despite this educational disadvantage, Black respondents consistently maintained a social capital advantage over time, suggesting that their social capital remains robust. Although Black individuals have historically faced systemic barriers to education, greater access to and engagement with education can provide additional opportunities to expand social networks, build transferable skills, and increase civic participation (25). Based on this long-standing evidence, the consistent social capital advantage of Black respondents in the face of persistent educational disadvantages and structural barriers would likely be strengthened and bring greater benefits with higher education attainment.

### 4.6 Limitations and strengths

Despite the stark racial differences in social capital observed in this study, it is important to acknowledge that our operational definitions and measures of social capital outcomes (“Contributions to Community” and “Community Involvement”) may not fully capture the breadth and complexity of social capital as described in the literature. Although not formally validated, these scales to measure social capital were developed and tested as part of the MIDUS study. Still, the use of non-validated, self-developed scales to measure social capital presents important limitations, particularly regarding construct validity and comparability across studies.

Social capital is a multifaceted construct shaped by cultural and systemic factors (25). Consequently, comparing social capital between racial groups requires careful consideration of the historical, systemic, and cultural contexts of each population studied. A notable limitation of the study is the limited racial heterogeneity and unbalanced racial group distribution, which may have affected the reliability of interaction terms and limited the precision of subgroup estimates when assessing differences in social capital trajectories over time. Additionally, our mixed-effects statistical model did not include a random slope, which would have allowed for individual-specific variability in the relationship between time (a categorical predictor) and outcomes (community contributions and involvement). This approach was not feasible due to the limited sample size and unbalanced racial group distribution across four levels of educational achievement. Furthermore, our model adjusted for only a limited set of confounders, which may have resulted in residual confounding and affected the interpretation of racial and educational disparities in social capital over time. Consequently, we assumed that the effect of time on outcomes was consistent across groups and chose a random intercept fixed-effects model. Although this assumption simplifies the model, it may oversimplify the relationship, especially for populations with diverse trajectories over time. However, given the lack of statistical significance for time in many instances and the small size of the Black sample, the random intercept model offered a more stable and parsimonious solution for the data.

Our study also had important strengths, including being one of the first to examine race and education longitudinally in relation to contributions and involvement in the community. We used longitudinal data from waves 1–3 of the MIDUS study, which allowed for a vigorous investigation into evolving patterns and trends over decades and strengthened the reliability of our findings. Second (to our knowledge), no previous study has used a single model to examine longitudinal changes of race and education related to community contributions and involvement. We used a single, mixed-effects statistical model with sophisticated statistical and imputation techniques to explore race and education together. This provided deeper insight into the relationships between the two variables and social capital. Third, we used the Disparity Assessment Framework by Ward et al. for assessing health disparities for a more rigorous approach. We found this to be critical for emphasizing the effect of size and visual plots, rather than relying on statistical significance alone. Unlike statistical significance (*p*-value), which only measures the likelihood that the results are due to chance, effect size and visual plots offer a more practical understanding of the magnitude of disparities in social capital across time, race, and educational level. This focus allows for a clearer interpretation of the real-world impact of disparities, beyond merely identifying whether differences are statistically significant.

### 4.7 Findings in the context of existing literature

Our finding that Black respondents demonstrated higher levels of social capital than their White counterparts align with existing literature. A few examples: two studies found that Black communities often foster strong communal ties and prioritize collective wellbeing, potentially as a response to systemic inequities (25, 36). Previous studies found that personal experiences, such as attending block parties or participating in Black Greek organizations and Black professional societies, were ways through which Black individuals foster sense of community (25, 36). Other studies indicated that personal experiences, cultural norms, historical factors, and community dynamics were pivotal in shaping the expression and impact of social capital within communities (37, 38). These expressions of social capital are less evident in White communities.

Our effect size as well as statistical significance also aligns with existing literature. Although the racial difference in community involvement was not statistically significant, incorporating effect size indicated that Black respondents may have a substantive advantage in certain dimensions of social capital. This confirms the importance of interpreting effect sizes alongside *p*-values to better understand the real-world impact of disparities (35). Because our study was one of the first to examine race and education longitudinally in relation to community contribution and involvement, as well as the first to do so using a single model, these findings are pioneering and fill critical gaps in literature.

### 4.8 Implications and future directions

This study advances our understanding of the complex relationships between race, education, and social capital over time. Longitudinal research is essential to understand these dynamics and address health disparities, especially in marginalized groups, which often face systemic barriers that reduce their social capital. Our findings have important implications for addressing social capital disparities—for both Black people and White people—to the benefit of both racial groups.

The racial and educational disparities we identified indicate a continuing need for policies and interventions to address systemic barriers to education and other resources. Future research should explore the mechanisms underlying the racial and educational disparities observed in this study. There is also a need for future studies to replicate these findings using validated and standardized measures of social capital. Other potential areas for further exploration include racial dynamics and culturally specific forms of social capital. The positive manifestations of social capital we observed should be explored and supported within the Black population.

Moreover, larger and more balanced samples are essential to allow for the use of advanced statistical techniques and ensure that findings are representative. Using random slope mixed-effects modeling would provide a more nuanced understanding of how social capital varies across individuals and groups over time. Incorporating qualitative methods would complement these quantitative findings by providing insights into the lived experiences that drive the patterns revealed in our study. In addition, research that fully integrates psychosocial and biological measures would enrich our understanding of how social capital impacts health, offering a more comprehensive and interdisciplinary perspective on the underlying mechanisms (39). Finally, the large effect sizes observed in the non-significant findings suggest that education’s influence on community involvement merits additional investigation.

## 5 Conclusion

Our findings highlight the importance of culturally sensitive approaches in social capital research. While we observed patterns of social capital across educational groups, particularly in domains such as contributions to community, some associations, especially those related to community involvement, did not reach statistical significance and should be interpreted with caution. These trends point to the need for continued exploration of how social capital manifests across racial and educational contexts rather than suggesting uniform effects. Recognizing the varied expressions of social capital is necessary to more accurately reflect the lived experiences of historically marginalized communities. Future research should build on these descriptive patterns to refine social capital measures and investigate their potential to inform strategies aimed at addressing health disparities. Through such intentional and inclusive efforts, we can deepen our understanding of social capital and its possible role in advancing health equity, especially for vulnerable populations.

## Data Availability

Publicly available datasets were analyzed in this study. This data can be found at: https://www.icpsr.umich.edu/web/NACDA/series/203.
